# Collective learning for resilience in Global South cities: a community-based systems mapping approach to integrated climate and health action

**DOI:** 10.3389/fpubh.2025.1582550

**Published:** 2025-05-19

**Authors:** Lidia Maria de Oliveira Morais, Elis Borde, Lambed Tatah, Meelan Thondoo, Feyisayo A. Wayas, Georgiana Gordon-Strachan, Charles Obonyo, Felix Assah, Tolu Oni, Waleska Teixeira Caiaffa, Leandro Garcia

**Affiliations:** ^1^Observatory for Urban Health in Belo Horizonte, Federal University of Minas Gerais (OSUBH/UFMG), Belo Horizonte, Brazil; ^2^MRC Epidemiology Unit, University of Cambridge, Cambridge, United Kingdom; ^3^Health of Population in Transition Research Group, University of Yaoundé I, Yaoundé, Cameroon; ^4^Research Centre for Health through Physical Activity, Lifestyle and Sport, Division of Physiological Sciences, Faculty of Health Sciences, Department of Human Biology, University of Cape Town, Cape Town, South Africa; ^5^Tropical Metabolism Research Unit, Caribbean Institute for Health Research, University of West Indies, Kingston, Jamaica; ^6^Centre for Global Health Research, Kenya Medical Research Institute, Kisumu, Kenya; ^7^Centre for Public Health, Queen’s University Belfast, Belfast, United Kingdom

**Keywords:** climate resilience, climate change, complex systems, Community-based System Dynamics, intersectorality, transdisciplinarity, Global South, urban health

## Abstract

**Introduction:**

Cities in the Global South face escalating climate change challenges, including extreme weather events that disproportionately affect marginalized populations and exacerbate health risks, such as non-communicable diseases (NCDs). Climate resilience, defined as the capacity to adapt and recover from climate-related events, requires intersectoral collaboration between governments and civil society.

**Methods:**

This study employs a Community-based System Dynamics approach, leveraging shared learning across four cities—Belo Horizonte (BH, Brazil), Yaoundé (Cameroon), Kingston (Jamaica), and Kisumu (Kenya)—through the Global Diet and Activity Research Network (GDAR). An implementation of the method in BH is detailed, examining drivers and interdependencies shaping community-based climate resilience strategies against heavy rainfalls.

**Results:**

In BH, findings highlight the interplay between urbanization risks, vulnerabilities, heavy rainfall, and NCDs, with visibility, resources, education, and training identified as critical intervention points.

**Conclusion:**

This study underscores the importance of aligning community action with public policy and highlights opportunities for collective learning and resilience-building for climate change in Global South cities.

## 1 Introduction

Cities in the Global South face escalating climate change challenges, including extreme weather events that disproportionately affect marginalized populations and exacerbate health risks, such as non-communicable diseases (NCDs) (1, 2). Rapid and unplanned urban growth, as often seen in cities of the Global South, has amplified inequalities with poor housing conditions and heightened vulnerabilities for large segments of the population, pushing people to live in hazard and disaster prone areas.

Building climate resilience in cities in the Global South is a pressing priority. Climate resilience refers to the capacity of communities, systems, and institutions to withstand, recover from, and adapt to the impacts of climate-related events such as floods, heavy rainfall, and heatwaves while maintaining essential functions (3–5). In response to these climate-related hazards and disaster events, local communities, governments, non-government organizations, and organized civil society have developed strategies to build resilience. For this paper, we refer to this group of actors as ‘community actors’ due to their impact and influence within communities affected by climate change.

Achieving sustainable and effective climate resilience strategies requires an intersectoral and systemic approach that integrates multiple sectors and perspectives. However, inefficient and non-existent dialog between community actors, each operating with fragmented perspectives and assumptions, hinders effective collective action (6). In addition, there is a knowledge-to-action gap on how these climate resilience strategies are built, reinforced, sustained, or weakened, and the intertwinement between actors and actions involved.

Prioritization and implementation approaches are rarely developed through genuine collaboration between governments and civil society. Civil society demands and pressures governments to act, yet public policy still falls short in recognizing that much of resilience-building occurs through collective action at the local level. Often led by local actors (5, 7) these initiatives draw on their deep understanding of community vulnerabilities, positioning them more effectively to mobilize resources and coordinate responses to climate events. A collaborative climate resilience strategy requires a shared understanding of the challenges at hand. This can be achieved through methodological approaches that promote dialog and collective learning, thus fostering collaboration and stronger and more sustainable resilience strategies (8, 9).

Moreover, the evidence base on the health consequences of climate-related events remains limited (10). These may include immediate injuries and deaths, as well as mid- and long-term effects such as the spread of infectious diseases and an increase in non-communicable diseases (NCDs) and their risk factors (1).

From a systems thinking perspective, understanding the interdependent chains of drivers and conditions that shape and sustain climate resilience strategies and NCD risks is essential for designing successful interventions. Systems thinking is an approach that looks not only at the immediate causes and consequences but at the structure and functioning of the system underlying the problem of interest (11, 12). It aims to understand how the various system’s components interact and co-evolve over time, contributing to both the emergence and persistence of the issue under investigation as a result of complex interrelationships and dynamics. It highlights principles such as interconnectedness, feedback loops, emergence, and a holistic perspective, recognizing that systems consist of interrelated parts that influence one another (13).

Climate resilience strategies and their drivers do not exist in isolation but interact dynamically with historical social inequities, environmental degradation, and the evolving urban landscape, forming complex, adaptive systems (6, 14–17). To involve community actors in the development of a shared understanding of the climate resilience system and developing insights crucial for successful interventions, Community-Based System Dynamics, a systems thinking approach, can be used to integrate participatory methods into system dynamics modeling (8, 9, 18). By assembling different community actors involved in local risk and disaster management and promoting a space for quality discussion and an in-depth dive into resilience strategies and their drivers, this approach provides a structured way to unearth and integrate diverse perspectives into policy planning and community action, fostering the collaborative networks necessary for long-term resilience. Community-Based System Dynamics allows for the co-creation of solutions that are grounded in local realities while supporting broader systemic change, being particularly valuable in Global South cities facing the syndemic of climate change, socio-economic inequities and health problems that creates unique challenges (19–21).

To explore community-based climate resilience strategies existent in the Global South and their intersection with prevention of NCD risks, we have leveraged global network for shared learning between 4 cities (Belo Horizonte, Brazil; Yaoundé, Cameroon; Kingston, Jamaica; Kisumu, Kenya), convened by the Global Diet and Activity Research Network (GDAR). Together, we developed a common approach to examine the interdependent chains of drivers and conditions that shape and sustain community-based climate resilience strategies while also aiming to optimize the generalizability of findings.

In this paper, we describe the shared learning of the methodological approach and how it was implemented in Belo Horizonte (BH), Brazil. We also share findings on BH local resilience strategies and their drivers for facing seasonal heavy rainfall aggravated by climate change, and its effects, including in relation to NCD risk factors. This work focuses on heavy rainfall, as it was identified by community actors in Belo Horizonte as the primary climate-related event with significant health impacts in the city.

## 2 Methods

### 2.1 Community-based system dynamics method adaptation and application

Our method consisted of three steps detailed in the sections below.

#### 2.1.1 STEP 1: Research team capacity building

GDAR researchers from Belo Horizonte, Yaoundé, Kisumu, and Kingston were trained and guided by a senior researcher (LG) with experience in Community-based System Dynamics and group model-building techniques. Three online training meetings were held over six months in 2023, encompassing theoretical and practical aspects of system thinking, Community-based System Dynamics, group model building, and the development of causal loop diagrams.

The first training session included an introduction to causal loop diagrams, the development of the individual interview guide (Step 3 - Phase 1, more details below), and training on the use of an online mapping and visualization software to record and represent individual mental models during interviews. Mental models are relatively long-lasting “explanatory schema that individuals use to explain the world and their interaction with it” (22). Mock practice of the process was conducted in the sites before proceeding to the interviews.

The second training session took place after the majority of interviews had been carried out, focusing on how to combine individual mental models and use an online mapping and visualization program to produce a preliminary version of the causal loop diagram that synthesized the interviewees’ individual mental models.

The third training session focused on how to prepare and conduct a group model-building activity following Community-based System Dynamics principles and practices, and focused on engaging the interviewees in the collective discussion and refinement of the preliminary synthesis causal loop diagram and on the facilitation of a shared understanding of the system. On top of the training sessions, the research team held regular meetings to discuss practical and conceptual issues that arose during the three steps and shared site-specific adaptations to data collection and analysis.

#### 2.1.2 STEP 2: Selection of participants

Participants were recruited through purposive sampling in collaboration with local partners, with snowball sampling used to further expand the pool of potential participants. Each site identified key community actors from various sectors engaged in local climate resilience strategies. This process included listing contacts from existing networks and identifying new connections that needed to be established. Invitations were extended either to individuals or to government sectors and civil society organizations, which were then asked to recommend a representative.

In BH, we invited all 26 participants from a previous GDAR stakeholder engagement meeting (1) in which the above described steps for invitation were executed. Nine participants responded and agreed to participate in individual interviews (Step 3 - Phase 1) and seven of them attended the participatory workshop (Step 3 - Phase 3). Participants, all adults with personal or professional ties to climate resilience strategies, came from local communities, government, and NGOs. The group consisted of men (4) and women (5), with representatives from climate-vulnerable communities (2), policymakers (5), and advocacy groups (2). The sessions were moderated by four members of the local research team. We considered that the diverse backgrounds and sectors of the 9 participants adequately represented the range of perspectives from the original group of 26 invitees.

#### 2.1.3 STEP 3: Building the CLD (4 phases)

##### 2.1.3.1 Phase 1: interviews to build individual mental models

The interviews aimed to surface individual mental models for each interviewee (22). To achieve this, a semi-structured interview guide divided into four parts was developed adapting the ‘Variable elicitation’, ‘Creating causal loop diagram from variable list’ and ‘Model review’ scripts at Scriptapedia (23). The full interview guide is available in Supplementary file 1 and below we present a summary of its parts:

Part 1 - Profile of the interviewee: interviewees answered questions about their profession; their role in the community/association/organization/institution they represent; their expertise on the subjects that would be dealt with during the interview; their experience with climate events and risks in their cities; and, their prior knowledge about systems thinking.

Part 2 - Identifying variables: interviewees were asked about their experience on climate events such as heavy rains and their consequences (floods/landslides events) in their cities, as well as to list and describe the most impactful strategies or activities that their community/association/organization/institution adopted as an adaptive response to these events. They were also asked to list factors/drivers that might facilitate and/or hinder these strategies and the potential effects of these strategies on individuals’ and population health, focusing on diet, physical activity, and NCDs. These variables were recorded by the interviewer on the mapping and visualization software (Mural), allowing the interviewee to see the list as it was built, adjusting in real time if needed.

Part 3 - Identifying relationships between variables: interviewees were instructed to indicate the relationships between the variables listed in part 2, and how these relationships are linked to population health, focusing on diet, physical activity, and NCDs. These links and their polarities were recorded by the interviewer on the mapping and visualization software (Mural) as a causal loop diagram, allowing the interviewee to see and shape the connections as they were built.

Part 4 – Causal loop diagram review: interviewees were then shown the causal-loop diagram that was developed and asked whether they would like to add or change anything on the diagram to better or more clearly represent the information they had shared.

In BH, Brazil, we conducted 9 interviews that led to the development of 9 causal loop diagrams representing the interviewees’ individual mental models. The interviews were conducted online by 2 interviewers (LM and EB).

##### 2.1.3.2 Phase 2: thematic analysis and development of preliminary synthesis causal loop diagram

The thematic analysis of the interviews’ content included both predetermined variables of interest to the research team (food, physical activity, health and NCDs, climate, inter-sectoral collaboration) informed by the research project focus and literature, and the variables elicited by the participants during the interviews. They were then consolidated into themes aiming to capture “semantic (explicit or overt) and latent (implicit, underlying; not necessarily unconscious) meanings” (24), that were used to create a consolidated list of variables to form the preliminary synthesis causal loop diagram.

In BH, the variables were organized in a spreadsheet by one researcher (LM), who first consolidated themes. Subsequently, a second reviewer (EB) cross-checked the themes against the interview material to ensure accuracy. The two researchers collaboratively synthesized the final themes, followed by a meeting with a third researcher (WC) to resolve any inconsistencies and achieve consensus.

Using the Kumu online platform, one researcher (LM) produced a preliminary version of the synthesis causal loop diagram with the variables and themes and the interconnections suggested during individual interviews. This diagram was discussed with other local research team members (EB, WC, LG) to check the synthesis diagram’s consistency in relation to the individual diagrams and the clarity of the variables and themes definitions.

##### 2.1.3.3 Phase 3: participatory workshops

Next, each GDAR site invited their local interviewees for a participatory workshop to share the preliminary version of the synthesis causal loop diagram and collectively refine variables, themes, and the connections between them. These workshops followed general principles and practices of group model building and Community-based System Dynamics (23), but the specific activities/agenda and mode of delivery (online or in-person) were adapted to the needs and participants in each site.

In BH, the participatory workshop was conducted in person on the morning of April 1st, 2024, and brought together seven out of the nine interviewees. The workshop started with a brief introduction to the systems thinking approach and of the phases taken to develop the preliminary synthesis causal loop diagram. In two small groups, facilitators guided participants in discussions about variables, themes, and the interconnections between them, based on the ‘Model review’ script at Scriptapedia (23). Finally, we brought the entire group together, facilitating discussions and fostering debate to build consensus on the proposed changes and the final causal loop diagram, including the identification of key feedback loops that helped to understand the functioning of the system.

##### 2.1.3.4 Phase 4: finalization of the causal loop diagram by the research team

The final step after the participatory workshop was for the research team in each site to make the remaining changes to the causal loop diagram after the inputs from the workshop to depict the participants’ shared understanding of community climate resilience strategies in their contexts.

In BH, after editing the causal loop diagram, we conducted an online one-hour meeting with seven of the nine interviewees on May 27th, 2024, for the research team to present the final version. Adjustments were made during the meeting, and the group agreed upon the final variables, themes, and connections.

### 2.2 Study setting

BH was planned in 1897 and has since grown well beyond its original contours, with an accompanying increase in population size. Currently, 2.3 million people live in the city, in a conurbation of 5 million people with neighboring cities (25).

BH is known for its innovative political environment, which in 1993 implemented a participatory budgeting pioneer plan, that is still an example worldwide (26). In its short lifetime, local actors and organized civil society have been important participants of the city’s decision-making and they are also necessarily part of the conversations about local climate resilience strategies.

Driven by public pressure on authorities, Belo Horizonte has developed robust food system policies and community urban transformations and social interventions. These include public services such as community kitchens and restaurants, formal and informal urban agriculture initiatives, regular markets, a comprehensive school meals policy (27), and a comprehensive urban transformation in *favelas* (slums) (28, 29). It has also embraced active living policies and infrastructure with cycling paths, parks, and closed streets during weekends to support physical activity (30). However, the distribution of these facilities is not always equal, with central (richer) neighborhoods having more access to these infrastructures.

In a previous GDAR stakeholder consultation event in BH, participants identified that while the city has been experiencing more frequent and intense heatwaves and droughts, the most impactful climate events are the heavy seasonal rains, leading to floods and landslides, and their consequences. This is because the urban fabric has grown rapidly and more haphazardly, populating areas that were originally in floodplains or hills that are prone to landslides (7, 31, 32). One consequence of this process is the city’s drainage system overloading.

BH has three hydrographic basins with many canalized rivers and streams (31). Heavy rainfalls are common over the summer months, occurring in concentrated bursts. The consequent floods and landslides result in loss of life and material losses of public and private goods. The heavy rainfalls of the 2020s were identified as particularly severe, along with significant events in 2018, 2014/2015, 2008, and 2003. These events often affect the same neighborhoods and areas (31, 32).

Residents of middle- to upper-class neighborhoods in central areas may experience temporary disruptions such as flooded streets, material losses (e.g., damaged vehicles), power outages, or, in rarer cases, structural damage to their homes. A notable example occurred in 2020 when the Santa Lucia dam partially collapsed due to severe flooding, causing damage to nearby buildings (31, 32). In contrast, residents in the city’s peripheries—often living in informal settlements on precarious hillsides with inadequate sanitation—face significantly greater risks. For these individuals, the seasonal rains bring the possibility of total loss of homes and possessions, exposure to life-threatening conditions, and waterborne diseases from sewage-contaminated floodwaters. Furthermore, infrastructure repairs in central areas tend to be addressed more quickly than in peripheral regions, exacerbating the vulnerability of marginalized communities and compounding the long-term effects of such events (33).

### 2.3 Ethics statement

All participants reviewed and signed informed consent forms to participate and authorized audio recording prior to the individual interview and their participation in the workshops. The project was approved by the Brazilian National Research Ethics Committee (CAAE 65706422.4.0000.5149) and the Cambridge Psychology Research Ethics Committee (Application No: PRE.2022.074, project NIHR133205).

## 3 Results

**Figure 1:**
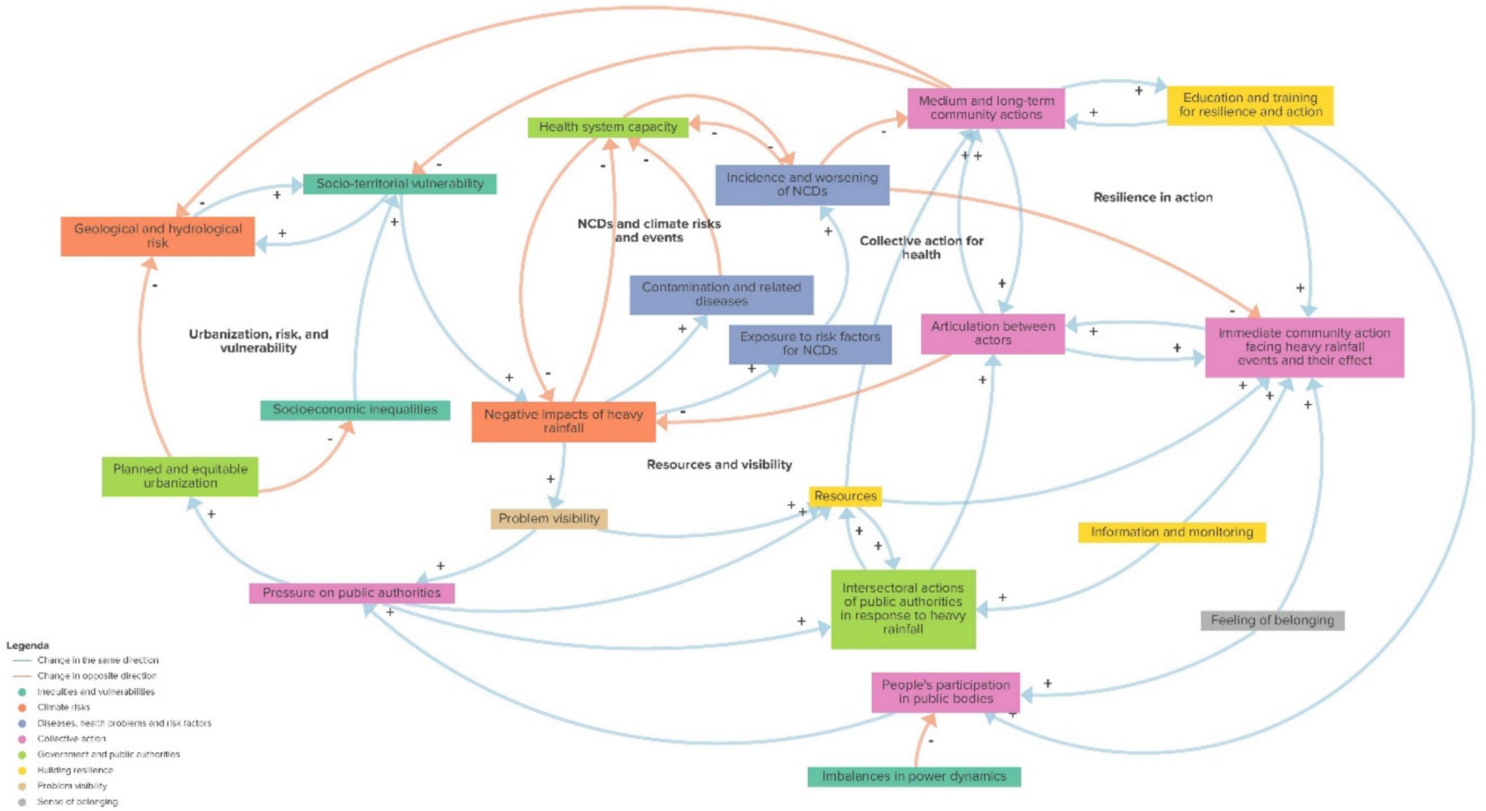
Causal loop diagram of heavy rainfall-related community resilience strategies, their drivers and conditions, organized by themes (colors), and five key feedback loops in Belo Horizonte, Brazil. Available at: https://embed.kumu.io/3bb57ca602f81426a1ebdbcf2a893d19.

The resulting causal loop diagram (Figure 1) encompasses 21 variables grouped into 8 themes (indicated by colors), interconnected by 31 positive causality relationships (indicating variables that change in the same direction) and 13 negative causality relationships (indicating variables that change in opposite directions), with multiple feedback loops, of which five are highlighted in the diagram (floating text). The diagram can also be visualized at: [https://embed.kumu.io/33c247eae039cb2cecd7ea369908f366].

A detailed description of the variables and themes is provided in Table 1.

**Table 1:** Causal loop diagram of heavy rainfall-related community resilience strategies: variables and themes description.

Theme	Variable	Definition
**Building resilience:** These variables are informational, subjective/social and/or material drivers and conditions that build and strengthen resilience strategies facing heavy rainfall events.	Information and monitoring	Information includes risk alerts and communication by public authorities, via telephone, messages and WhatsApp; press and social networks articulated actions; installation of sirens and alerts in risk areas; and rain alert centers, among other strategies. In monitoring it is included the city’s risk and disaster management group seasonal actions; the civil defense technical visits, periodic inspections, monitoring and reports in flood risk areas, technical support for preventing food safety risks, and territorial actions, carried out together with communities; the pluviometric and fluviometric stations that measure the level of rivers and streams with satellite image; the communication channels with the Belo Horizonte Urbanization and Housing Company (URBEL) regular inspection for landslides risk assessment; the epidemiological surveillance organized within the Universal Health Systems (SUS); and the mapping and periodic technical visits to monitor territories, house materials, risk areas, potential for agriculture, among other indicators, developed by the secretariat of social assistance, food security and citizenship and other government bodies.
	Resources	Include financial, human and material contributions to resilience actions, which, in general, will help build capacity for action. As for financial resources, we include public budget financing or inputs from their sector. Human resources often mean voluntary work, people willing to help in the field after disasters and support victims in various ways, help cleaning, cooking and the civil defense work, for example. Material resources can include donations, carriage of goods to be distributed to victims or the victims belongings and furniture after floods and landslides.
	Education and training for resilience and action	Both formal practices of school education and academic research, as well as informal educational and training practices for community dwellers and government professionals. These initiatives may include safety procedures and guidance on how to act when facing heavy rainfall, floods and landslides; how to access available public services; how to manage waste disposal; and also capacity building of local multipliers.
**Climate risks:** It encompasses consequences of heavy rainfall in relation to the geographic and material conditions specific to the territories.	Negative impacts of heavy rainfall	It encompasses both immediate and long-term effects of heavy rainfall such as the loss of lives and the deterioration of built environments and livelihoods, and their consequences.
	Geological and hydrological risk	Factors that may augment risk of floods and landslides in situations of intense rain, such as vertical cut of the ravine, water percolating in the ground, soil sealing and leaching, channeled river network, among others.
**Collective action:** Group action autonomously organized by civil society actors	Immediate community action facing heavy rainfall events and their effect	Includes voluntary action to support victims with cooking, welcoming to temporary shelter, offering mental health and caring support, offering information on adequate services and places to appeal in case of disasters.
	Articulation between actors	Effective coordination combines resources and efforts to build resilience when facing extreme climate events, both in terms of materials and personnel. This coordination allows complex and multi-disciplinary endeavors.
	Pressure on public authorities	Civil society acting to push public authorities and governments to recognize/implement their demands.
	People’s participation in public bodies	Official instances of participation such as councils and conferences, participatory budgeting, public hearings in the city council, among others.
	Medium and long-term community actions	Include community organizations and associations, advocacy groups, self-organized initiatives that last through time, with various purposes.
**Diseases, health problems and risk factors:** health outcomes, their drivers and conditions	Exposure to risk factors for NCDs	Include degraded urban environment, poor diet and physical activity, mental health conditions among others.
	Incidence and worsening of NCDs	Incidence and worsening of NCDs.
	Contamination and related diseases	Water and soil contamination after floods, which may put people in direct contact with sewage water and solid waste, spreading infectious diseases such as cholera and leptospirosis, and increasing the risk of accidents with venomous animals. Furthermore, mosquitoes that transmit arboviruses find ideal conditions for breeding, increasing the incidence of diseases such as dengue, zika and chikungunya, among others. There is also the risk of exposure to industrial components and chemical contaminants due to floods in industrial areas, for example, which is not a main component of this system, but should be taken into consideration.
**Government and public authorities:** Medium and long-term structures, services and functions of public authorities, as well as their immediate capacity for mobilization and action in face of situations of extreme climatic events.	Intersectoral actions of public authorities in response to heavy rainfall	Include articulations between public authority bodies, secretariats, ministries, executive and legislative powers, among others.
	Planned and equitable urbanization	Includes urban planning and interventions that are oriented by or intend to promote equity, and help to balance social and economic inequalities, and promote social inclusion. This type of urbanization, ideally, includes the interests of the population, is participatory and transparent. This approach to urbanization contrasts with the accelerated urbanization processes focused on profit that exacerbates social inequities and changes local environments, in general, to warmer and less permeable conditions and, therefore, more prone to flooding and soil degradation.
	Health system capacity	The ability of a health system to effectively deliver quality health services and respond to the health needs of a population.
**Inequities and vulnerabilities:** Longstanding structural and social inequalities faced by communities in the global south flagging the uneven distribution of risks and disasters associated with climate events.	Socio-territorial vulnerability	Social inequalities associated with territorial vulnerabilities, such as living in slopes and riverbanks, in the outskirts of urban development areas, or in slums (favelas) within the urban areas, with poor housing conditions.
	Socioeconomic inequalities	Inequalities related to socio-economic characteristics such as income, education, race, gender, age, among others.
	Imbalances in power dynamics	The unequal distribution and exercise of decision-making power among social actors, shaped by structural and systemic factors.
**Problem visibility** (homonymous variable definition)	Problem visibility	The dissemination of information through social networks, traditional media such as radio and television, and popular mobilizations in the streets.
**Feeling of belonging** (homonymous variable definition)	Feeling of belonging	This feeling is related to the construction of an identity with the place in which one lives, whether the neighborhood or the house, and the social networks that are established in these neighborhoods and communities. It enhances empathy and willingness to help community fellows.

In this section, we describe the themes and the variables organized within them, indicating their relationships with other variables and themes. When a variable from a different theme is mentioned within a theme subsection, the original theme is described in parentheses.

### 3.1 Themes and variables

#### 3.1.1 Inequities and vulnerabilities (dark green variables)

The theme “Inequalities and vulnerabilities” encompasses the following variables: socioeconomic inequalities; socio-territorial vulnerability; and imbalances in power dynamics and their effects on resilience strategies.

According to the participants, more ‘Socioeconomic inequalities’ lead to more ‘Socio-territorial vulnerability’. This connection is made because socioeconomic conditions can create barriers to land access and the granting of housing rights. As a result, people of low socioeconomic status often live in high-risk areas or housing with poor structural quality, such as slopes or riverbanks prone to seasonal flooding (31–33).

If no people were affected, it would just be an ordinary weather event. (Workshop participant, representatives from climate-vulnerable communities)

Likewise, ‘Socio-territorial vulnerability’ increases the ‘Negative impacts of heavy rainfall’ (Climate risk), as the most significant impacts of rains, floods, and landslides occur in these high-risk areas or housing with poor structural quality, suffering more from the negative impacts of heavy rainfall and having limited access to health or civil defense services, both essential for the immediate response to heavy rain events. Notably, central and higher socioeconomic status areas of the city receive significantly more attention and resources for urban planning, climate disaster prevention, and correcting damages when necessary (31–33). For example, the rapid road reconstruction in high socioeconomic areas of Belo Horizonte following the 2020 floods contrasts with the slower recovery efforts in lower socioeconomic status areas, as participants highlighted.

Also, the higher the ‘Imbalances in power dynamics’, the lower the ‘People’s participation in public bodies’ (Collective action). Community actors pointed out that the more hierarchical the government is, the more difficult it is to access government institutions and participatory bodies, even if they are set out in legislation. The same applies if there are powerful market institutions or any other actor who creates significant power imbalances through their economic capital. Information, education, and capital are all factors that contribute to unequal access and may discourage participation in conferences, committees, forums, audiences, and other public actions and participation.

#### 3.1.2 Climate risk (orange variables)

The “Climate risk” theme included: geological and hydrological risk and negative impacts of heavy rainfall.

One participant highlighted:

The concept of risk can be understood as a three-factor equation. The first factor is the threat, which can be natural or human-induced. The second is the vulnerability of the ecosystem that is exposed to these threats. The third, equally important, is exposure, referring to how close a threat is to a vulnerable environment. Together, these factors determine the overall risk faced by the ecosystem. (Workshop participant, policymaker)

A higher ‘Geological and hydrological risk’ was indicated to lead to higher ‘Socio-territorial vulnerability’ and vice versa. This means that the more risk a territory is exposed to, the more vulnerable it is and its people. One participant discussing the impacts of floods in vulnerable communities explained:

They lose everything, everything. It’s difficult to rebuild, to start over when the person’s shed collapsed, the stove went away, the bed broke, the closet fell apart, and that’s their life built over the years, and it gets lost in one night. (Workshop participant, advocacy group)

The higher the ‘Negative impacts of heavy rainfall’, the higher the ‘Contamination and related diseases’ (Diseases, health problems, and risk factors). In the medium and long term, heavy rainfall may also increase the ‘Exposure to risk factors for NCDs’ (Diseases, health problems, and risk factors) because of changes in living standards, diet, physical activity, or mental health.

The more the ‘Negative impacts of heavy rainfall’, the greater the ‘Problem visibility’, since a large volume of water, extensive destruction of public property, and significant loss of life may better attract attention and public outcry, gaining more space in the media, for example.

Additionally, higher ‘Negative impacts of heavy rainfall’ was linked to lower ‘Health System capacity’ (Government and public authorities) since the health system may become overwhelmed with lower capacity in both material resources and human care infrastructure due to the acute (e.g., injuries, infectious diseases) and chronic (e.g., mental health conditions) health effects of heavy rainfall.

#### 3.1.3 Diseases, health problems, and risk factors (purple variables)

The theme “Diseases, health problems and risk factors” refers to health outcomes sensitive to the occurrence of heavy rainfall in Belo Horizonte and comprises 3 variables: exposure to risk factors for NCDs; incidence and worsening of NCDs; and contamination and related diseases.

Increased ‘Exposure to risk factors for NCDs’ leads to a higher ‘Incidence and worsening of NCDs’. As mentioned by participants, living in risky situations produces harmful effects on mental health, such as difficulty in sleeping, anxiety, and fear. Some individuals, for instance, may choose to avoid going to work if rain has been forecasted out of fear of leaving their children unattended. As highlighted by workshop participants, this behavior can escalate into broader economic and social challenges.

The greater ‘Incidence and worsening of NCDs’, limits both ‘Immediate’ and ‘Medium and long-term community actions’ (Collective action) in response to heavy rainfall and its effects. According to participants, if people are physically and/or mentally ill, they have a lower capacity for action and coordination in response to climate events. For example, they are less likely to show interest or dedicate time and willingness to engage in emergency rescue and support efforts for victims of climate disasters. Additionally, sustaining their participation in community organizations, initiatives, or advocacy actions with public authorities becomes increasingly challenging.

All of the mentioned health effects weaken the ‘Health care systems capacity’ (Government and public authorities) by overloading it and thus (re-)producing health inequalities.

#### 3.1.4 Collective action (dark pink variables)

The “Collective action” encompasses five variables: immediate community action facing heavy rainfall events and their effects; medium and long-term community actions; pressure on public authorities; articulation between actors; and people’s participation in public bodies.

The greater the ‘Immediate community action facing heavy rainfall events and their effects’, the stronger the ‘Articulation between actors’. Greater mobilization encourages more individuals and organizations to unite around a problem, fostering collaboration and coordination among actors. A more extensive and interconnected network becomes more vigorous, as it can effectively leverage the resources and expertise of diverse partners, community associations, and local initiatives.

Likewise, the stronger the ‘Medium and long-term community actions’, the stronger the ‘Articulation between actors’ and the ‘Education and training for resilience and action’ (Building resilience), for the same reasons as above. Local organizations make education and training viable in local communities by hosting, mobilizing, offering, and replicating projects and programs to build resilience strategies. Besides, the process of action and participation itself is pedagogical for those who participate and leads to engaging more people interested in learning and participating in a virtuous feedback cycle.

Moreover, the stronger the ‘Medium and long-term community actions’, the lower the ‘Geological and hydrological risk’ (Climate risk) and the lower the ‘Socio-territorial vulnerability’ (Inequities and vulnerabilities). Through persistent and continuous efforts driven by popular demand and participation, these actions can potentially transform urban structural and systemic conditions. By doing so, they promote improvements in living standards and housing conditions for the most vulnerable populations. Additionally, these actions can advocate for enhanced public assistance for individuals affected by climate change-related disasters, thereby reducing vulnerabilities and fostering resilience.

The greater the ‘People’s participation in public bodies’, and here we refer to official instances of civil participation such as councils and conferences, participatory budgeting, and public hearings in the City Council, among others, the greater the ‘Pressure on public authorities’. Occupying these official spaces and having community voices heard may increase trust in the actions of public authorities. In turn, this can lead to more informed and adaptive responses from public management in the face of disasters, improving the quality of care provided and helping to prevent the re-victimization of those affected by climate-related disasters.

A well-structured and continuous ‘People’s participation in public bodies’ helps public management to consider the vulnerable population as partners, to be heard and active subjects of the solution, creating representative approaches that welcome diversity. People’s participation in public bodies supports public management in recognizing vulnerable populations as partners—active agents to be heard and engaged in the search for solutions. This represents an ideal-typical outcome, as described by participants within a well-functioning system. However, persistent power imbalances and conflicts can undermine this potential, as also reflected in the theme of “Imbalances in power dynamics.

Increased ‘Pressure on public authorities’ leads to increased ‘Resources’ (Building resilience), ‘Intersectoral actions of public authorities in response to heavy rainfall’ (Government and public authorities), and ‘Planned and equitable urbanization’ (Government and public authorities). Public authorities allocate resources and act proactively influenced by pressure from various social actors, who contribute by informing, criticizing, pressuring, and helping the public authorities act in the population’s interests. It is also based on this pressure that intersectoral actions are mobilized, which combine various bodies, departments, and work fronts of public authorities, and that urbanization projects and interventions incorporate approaches to equity.

Moreover, the greater the ‘Articulation between actors’, the lower the ‘Negative impacts of heavy rainfall’ (Climate risks) since it allows better monitoring of the displaced or injured individuals, assessing site risks to determine when families can safely return home, and providing essential support services like psychological care, nutrition, and shelter. A more diverse network can also raise resources and mobilize actions with public authorities or the media, augmenting the response potential. Therefore, the greater the ‘Articulation between actors’, the greater the ‘Immediate community action facing heavy rainfall events and their effects’ and the ‘Medium and long-term community actions’, as discussed previously.

#### 3.1.5 Government and public authorities (light green variables)

The theme “Government and public authorities” includes the variables: planned and equitable urbanization; health system capacity; and intersectoral actions of public authorities in response to heavy rainfall.

The more ‘Intersectoral actions by public authorities facing heavy rainfall’, the greater the ‘Articulation between actors’, augmenting the likelihood that their actions will mobilize sufficient resources to build and implement effective resilience strategies in response to climate disasters. Resources also tend to be more rationally, effectively, and efficiently used if accompanied by more intersectoral actions, creating a virtuous feedback loop between the two variables.

As a function of public authorities responding to people’s needs, the more ‘Planned and equitable urbanization’, the lower the ‘Socio-economic inequalities’ (Inequities and vulnerabilities) and the lower the ‘Geological and hydrological risk’ (Climate risk). In Belo Horizonte, it may include policies such as the *Green-Blue Trama* or the *Multicolor Trama* (34), public transport systems that align risk prevention (by promoting active mobility and urban agriculture), and adaptation actions (encouraging agroecological and nature-based practices to reduce flooding risk and contribute to food security). Workshop participants highlighted the need for urban design and planning to actively promote equity in the form and function of public spaces and to preserve the connections between the natural environment (such as water beds and green areas) and the various daily activities and ways of life of city inhabitants.

As expected, greater ‘Health system capacity’ correlates with reduced ‘Negative impacts of heavy rainfall’ (climate risks) and a lower ‘Incidence and worsening of NCDs’ (NCDs, health problems, and risk factors). With a well-established capacity, the effectiveness of primary care increases, enhancing its potential to prevent NCDs and reduce fatalities during extreme weather events, offer protection for vulnerable groups (such as children, the older adult, and women), and maintain the health surveillance infrastructure in continuous operation. If the ‘Health system capacity’ weakens, the incidence and worsening of NCDs increase as the system cannot meet population demands, potentially leading to treatment interruptions. Additionally, the ‘Negative effects of heavy rains’ may intensify, further exacerbating vulnerabilities and creating a vicious cycle of declining health conditions.

#### 3.1.6 Building resilience (yellow variables)

The “Building resilience” theme encompasses the following variables: resources; education and training for resilience and action; and information and monitoring.

The more ‘Resources’, the more ‘Immediate community action facing heavy rainfall and their effects’ (Collective action), ‘Medium and long-term community actions’ (Collective action), and ‘Intersectoral actions of public authorities in response to heavy rainfall’ (Government and public authorities).

The more ‘Education and training for resilience and action’, the more ‘Immediate community action facing heavy rainfall and their effects’ (Collective action), ‘Medium and long-term community actions’ (Collective action), and ‘People’s participation in public bodies’ (Collective action). Education and training to face climate risks and disasters in communities may be led by community associations or government bodies, such as the “Civil Defense in Schools” project, which promotes learning for understanding and coping with climate disasters. It is of note that all collective action variables are directly or indirectly (via another Collective action variable) influenced in the same direction by the ‘Education and training for resilience and action’ variable. This is because, according to community actors, education and training for resilience involve creating favorable environments that foster people’s political development, engagement in community activities and official participatory spaces. These environments, in turn, create a positive feedback loop, enabling further learning and training for those involved. Although of ultimate importance, participants highlight that collective action does not dismiss public policies and government action for climate resilience strategies.

The more ‘Information and monitoring’, the more ‘Immediate community action facing heavy rainfall and their effects’ (Collective action), and the more ‘Intersectoral actions by public authorities in the face of rains’ (Government and public authorities).

#### 3.1.7 Problem visibility (golden variable)

This theme contains only one homonymous variable, ‘Problem visibility’. This highlights the importance of being seen and actively documenting, sharing, and amplifying events and actions to strengthen the resilience system in response to climate events.

Visibility helps set the agenda of public authorities and organizations at local, national, and international levels and may guide the attention and direct actions of authorities. Consequently, the greater the ‘Problem visibility’, the greater the ‘Pressure on public authorities’ (Collective action), and the greater the ‘Resources’ (Building resilience) allocated.

Greater visibility can drive action, attract interest, and mobilize resources. However, as community actors have emphasized, it is essential to ensure that visibility is not exploited as a political tool, a source of misinformation, or reduced to sensationalism in the media. Such misuse may divert attention and efforts away from critical points of action, spreading misleading information that can create a detrimental effect on the system. This, in turn, may weaken public pressure on authorities and hinder allocating necessary resources to address the problem effectively.

#### 3.1.8 Feeling of belonging (gray variable)

This theme contains only one homonymous variable, ‘Feeling of belonging’.

The feeling of belonging to a community increases the interest and availability for ‘People’s participation in public bodies’ (Collective action) and the ‘Immediate community action facing heavy rainfall and their effects’ (Collective action). It is a powerful motivator for individuals to engage and maintain their involvement in various collective actions, whether striving for improved living conditions in their communities or confronting a common threat. This engagement can manifest in debates and actions within official participatory spaces or in implementing emergency resilience strategies to address climate disasters.

### 3.2 Key feedback loops

Feedback loops are key structures to understand how the internal dynamics of a system determine the way it functions. There are two types of feedback loops:

In balancing feedback loops, behaviors or events inside the loop counter one another, resisting changes in one direction over time. In reinforcing feedback loops, behaviors or events inside the loop reinforce one another, amplifying the effect of the process over time (35).

Here, we highlight five feedback loops for their central role and interconnectedness within strategies and related drivers.

**Figure 2:**
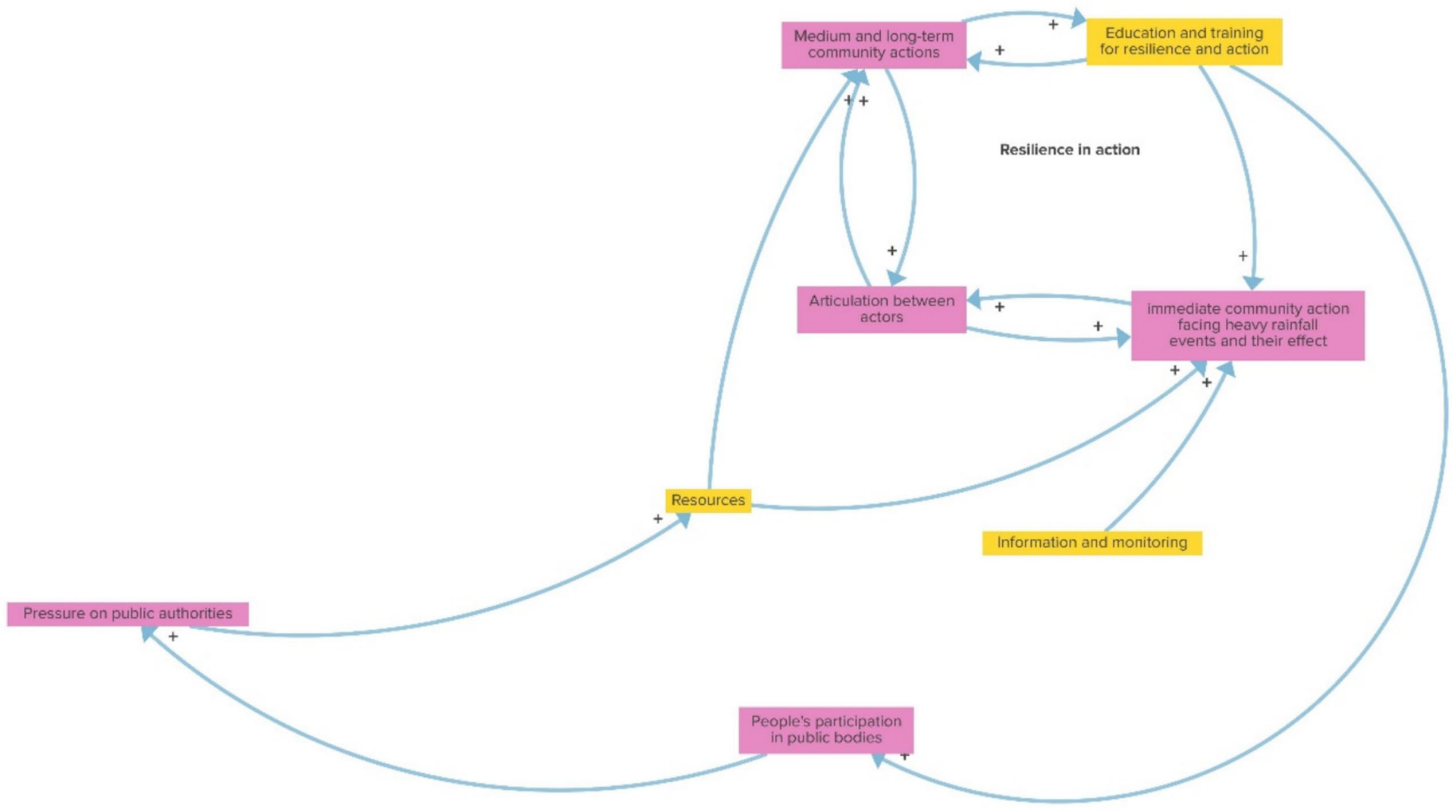
Education and action (reinforcing loop).

In the EDUCATION AND ACTION reinforcing loop, as shown in Figure 2, ‘Building resilience’ and the ‘Education’ themes appear very strongly connected. Increased education and training for resilience lead to heightened community action, which subsequently enhances intersectoral coordination and further educates the community, creating a cycle of resilience-building actions. Increasing education and training on resilience and action increases immediate community action in the face of heavy rainfall and its effects, articulation among actors, and medium and long-term collective actions term, which, in turn, influence education and training for resilience and action in the same direction again, forming a positive reinforcing feedback loop. From a broader perspective, an encompassing loop includes education and training, also augmenting people’s participation in public bodies and consequently, pressure on public authorities, promoting the allocation of resources, and both immediate and mid and long-term community actions.

**Figure 3:**
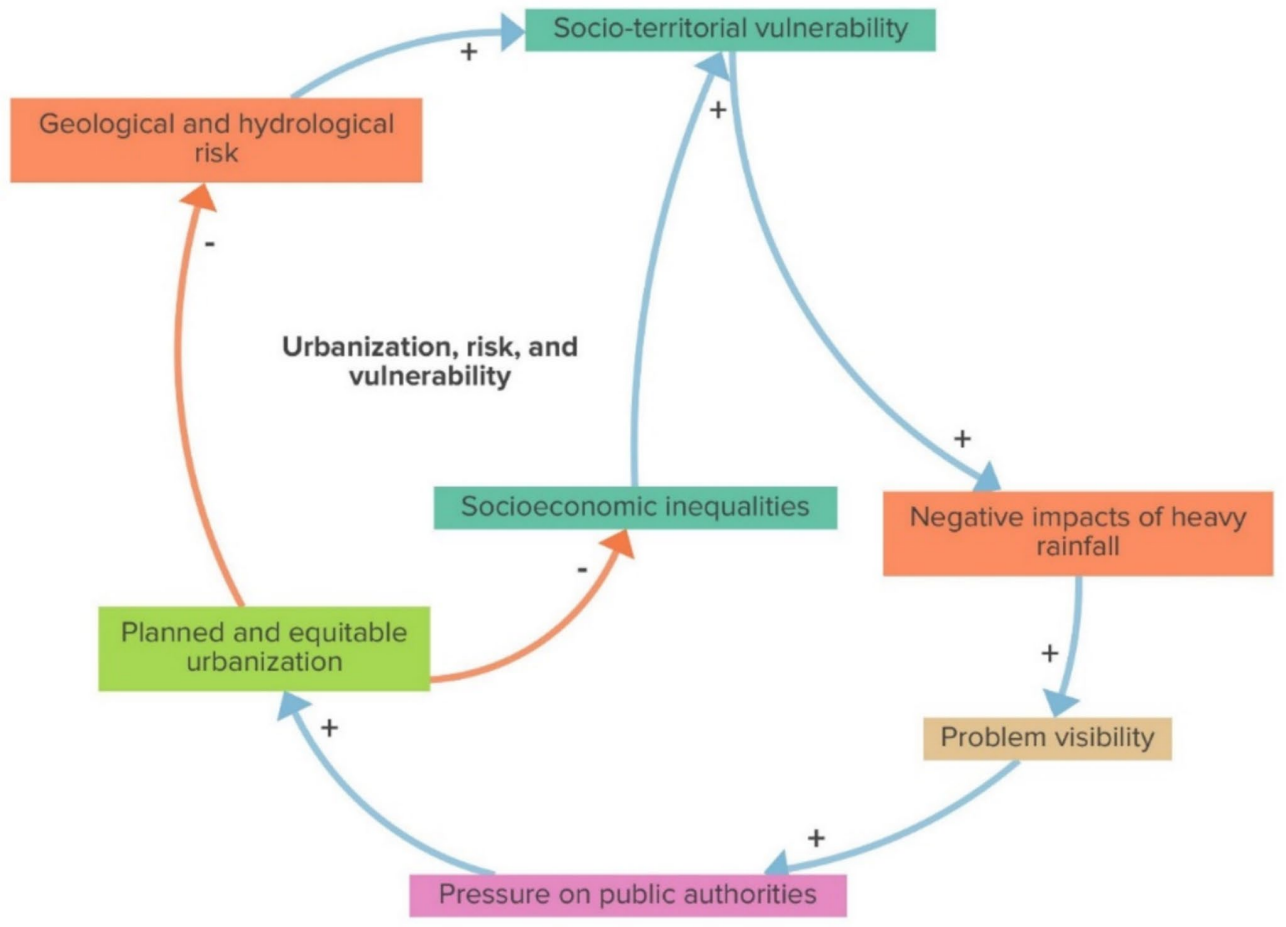
Urbanization, risk and vulnerability (balancing loop).

The urbanization, risk, and vulnerability balancing loop, as shown in Figure 3, indicates that planned, equitable, and environmentally sustainable urbanization processes diminish geological and hydrological risk and socio-territorial inequalities. Both/either variables decrease, and social and territorial vulnerabilities decrease, followed by a decline in the negative impacts of heavy rainfall. This sequence of events reduces problem visibility and, thus, relieve pressure on public authorities for new urbanization actions.

**Figure 4:**
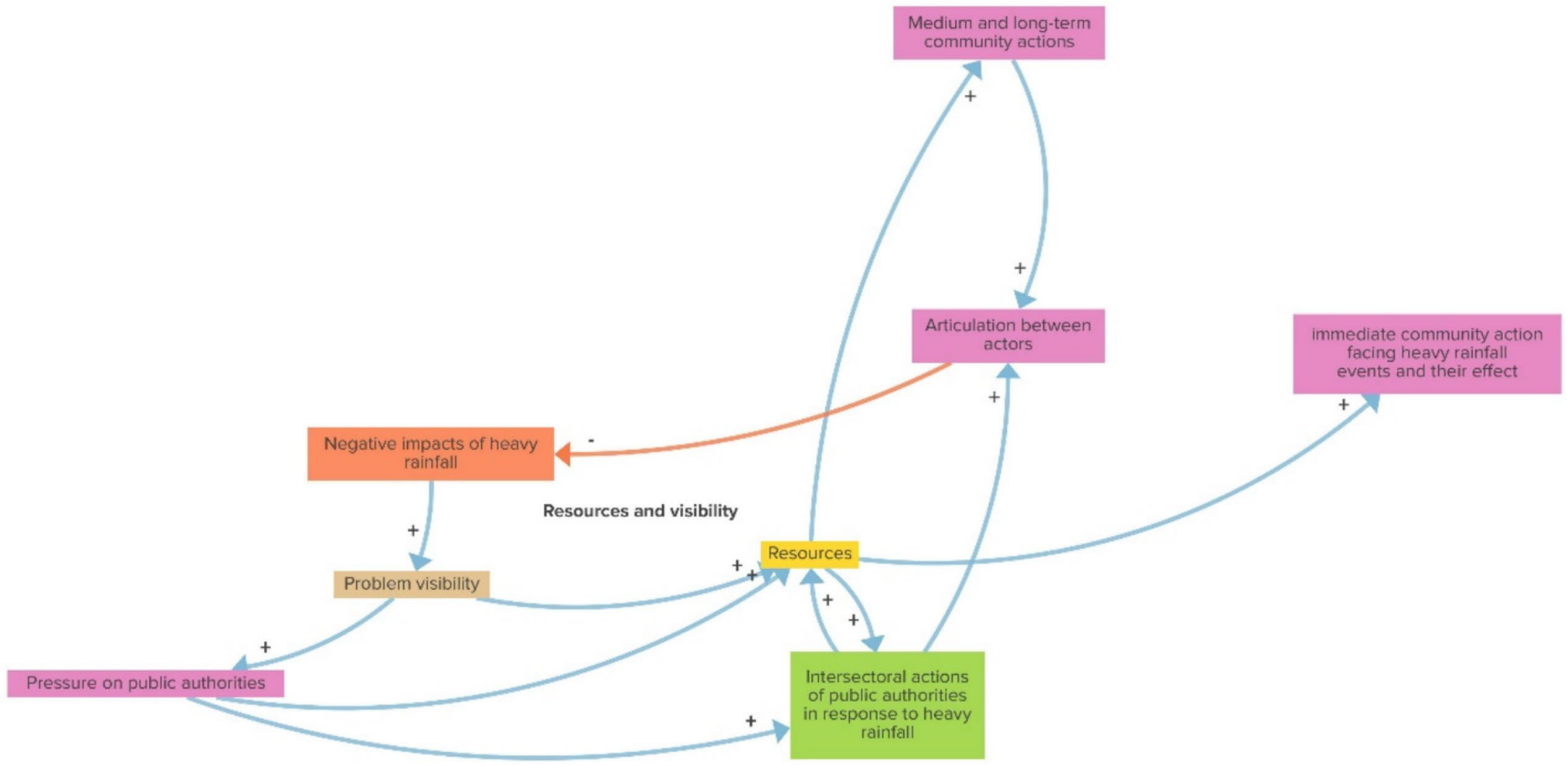
Resources and visibility (balancing loop).

In the resources and visibility balancing loop, as shown in Figure 4, the more ‘Negative effects of heavy rainfall’, the more ‘Problem visibility’, which leads to more ‘Resources’ and more ‘Pressure on public authorities’. More resources tend to increase both ‘Immediate’ and ‘Medium and long-term community actions’, as well as ‘Intersectoral actions of public authorities’, which is also increased based on more ‘Pressure on public authorities’. Both ‘Resources’ and ‘Intersectoral actions’ reinforce the articulation between actors, which, then, may mitigate the ‘Negative effects of heavy rains, also reducing the ‘Problem visibility’ and, potentially, future ‘Resources’ and actions/articulations in a scenario where the negative impacts are not as visible anymore.

**Figure 5:**
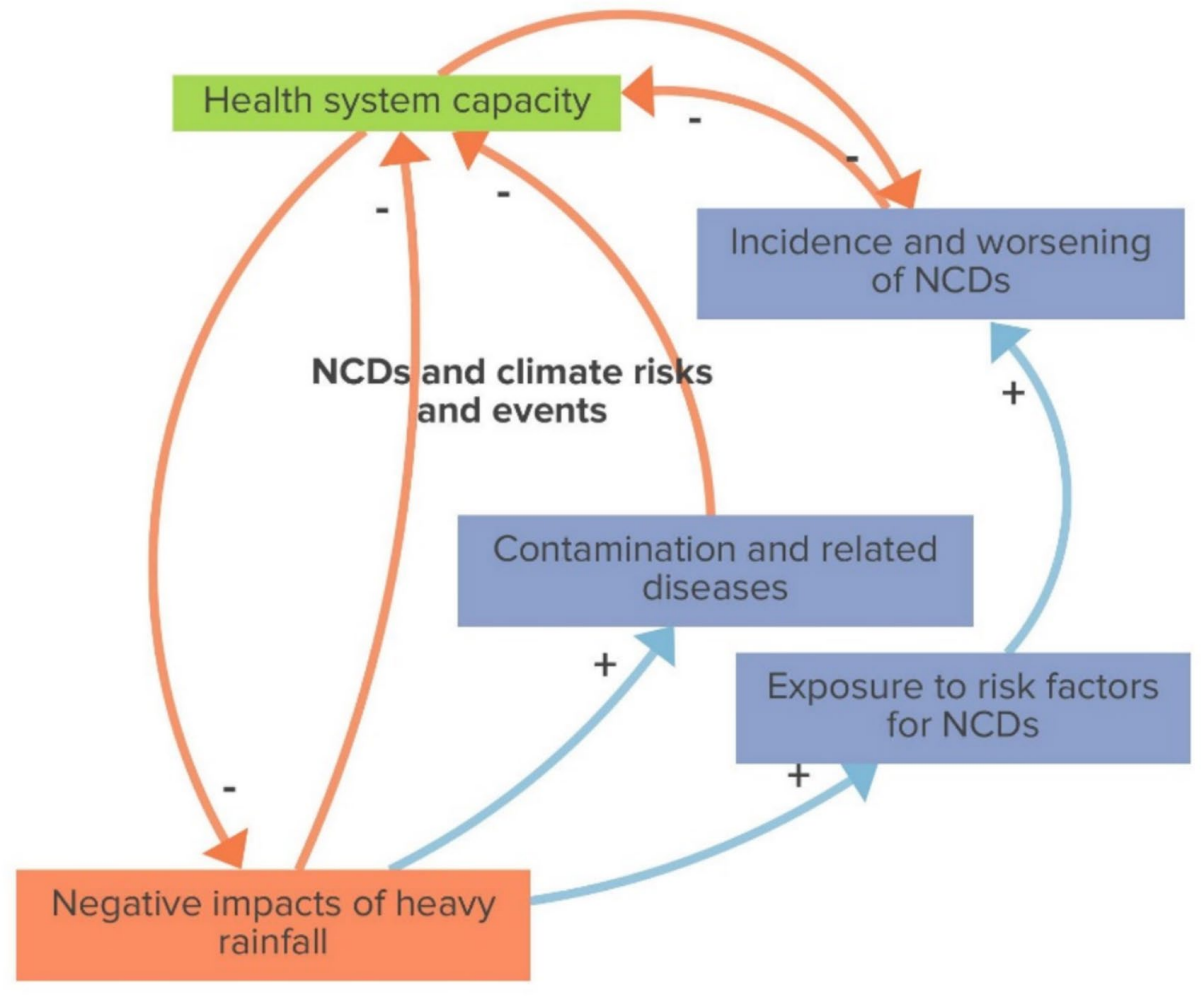
NCDs and climate risks and events (reinforcing loop).

The NCDs and climate risks and events reinforcing loop, as shown in Figure 5, indicate that the greater the negative impacts of heavy rainfall, the higher the incidence of health outcomes such as contaminations, related diseases, and increased exposure to risk factors for non-communicable diseases (NCDs), leading to a rise in NCD cases. This surge strains the health system’s capacity, further exacerbating its inability to address the negative impacts of rainfall, thereby perpetuating a vicious cycle of worsening health outcomes and reduced system resilience.

**Figure 6:**
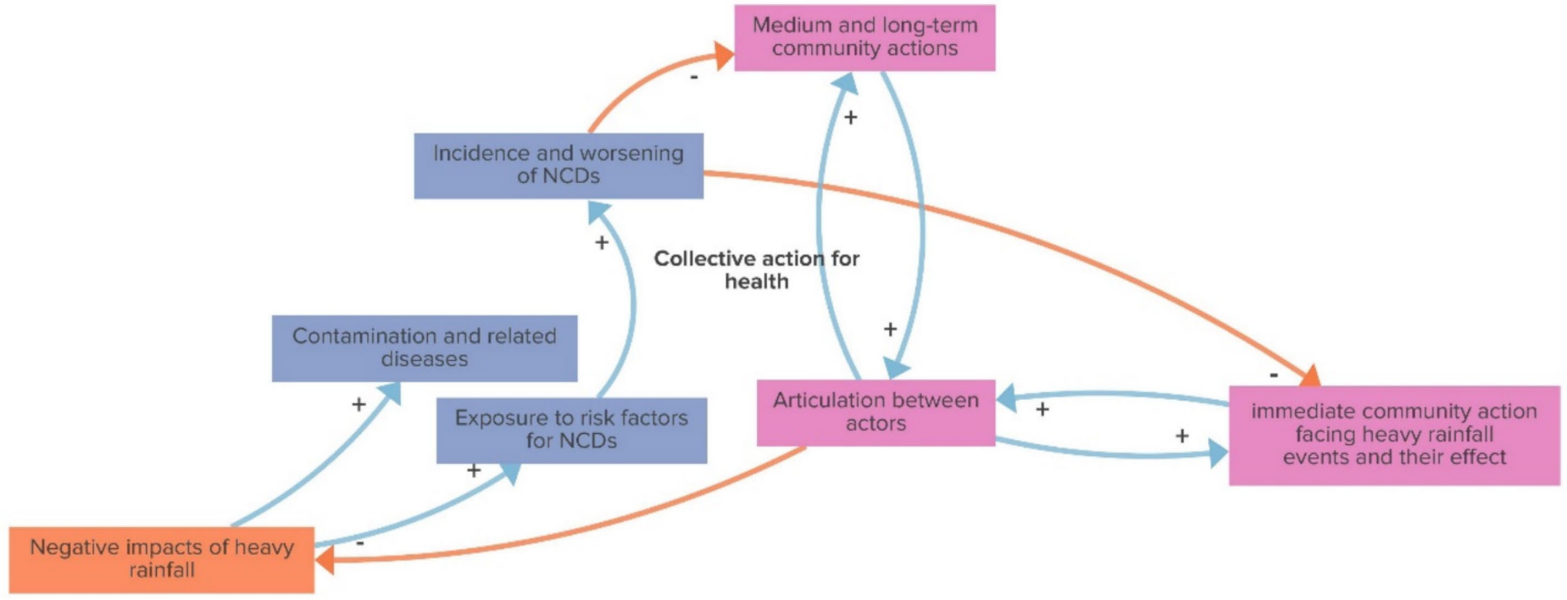
Collective action for health (reinforcing loop).

The collective action for health reinforcing loop, as shown in Figure 6, highlights that the more severe the negative effects of heavy rainfall, the greater the health impacts, including contaminations, related diseases, and increased exposure to risk factors for non-communicable diseases (NCDs), leading to a higher incidence of NCDs. As NCD incidence rises and health conditions worsen, individuals have a reduced capacity to engage in both immediate and longer-term community actions. This diminished engagement weakens the articulation and collaboration between actors, undermining the community’s collective capacity for action. This reduced articulation, in turn, amplifies the negative effects of heavy rainfall, perpetuating a self-reinforcing cycle of vulnerability and diminished resilience.

## 4 Discussion

This study explored the interdependencies among various drivers and conditions influencing BH climate resilience strategies for heavy rainfall and their influence on health and specifically NCD risks, based on the shared understanding of this complex problem among the local community actors.

Cities in the Global South, including Belo Horizonte, have experienced exclusionary urbanization processes that have deepened segregation within urban areas, resulting in stark inequities in resource distribution and access to public services. These conditions fundamentally shape climate resilience strategies in the city.

As highlighted by the Urbanization, risk, and vulnerability loop, the ‘Climate risk’ variables are heavily connected to ‘Inequalities and vulnerabilities’. This is so because climate risks and the impacts of climate events are inequitably distributed across different regions and populations of the city. The people who mainly suffer the consequences of heavy rainfalls and the consequent floods and landslides are populations already in socioeconomic vulnerability. More affluent neighborhoods in BH, such as Buritis, Santa Lúcia, and Mangabeiras, also face significant challenges, such as soil sealing and frequent flooding due to the soil conditions and poorly planned human interventions, exacerbated by rapid urbanization. Nevertheless, the events gain much more visibility and faster repairs than in suburban or slum areas of the city.

Another critical point raised by community actors is that vulnerable populations often have a deep attachment to their homes and possessions, which were acquired through significant effort and sacrifice. This strong emotional and material connection can heighten their resistance to evacuate and leave their belongings behind during high-risk situations.

Many families, even having gone through floods and landslides that have put them at risk in previous years and having been removed/relocated from their homes, would end up returning to their old houses or neighboring areas for a feeling of belonging, an unsuccessful relocation process or other various reasons, as described by interview and workshop participants.

The heightened climate risk for populations living in vulnerable areas is further exacerbated by the effects of heavy rainfall, as depicted in the NCDs and climate risks and events loop. This loop describes how the negative effects of heavy rainfall directly affect multiple health outcomes in the short- (deaths, contamination, and related diseases), medium- and long-term (risk factors, incidence, and worsening of NCDs).

In BH, community actors indicated that in the medium and long term, a sensitive risk factor for ‘Incidence and worsening of NCD conditions’ and ‘Contamination and related diseases’ is the availability and quality of food. This, in turn, is affected by the dynamics of rainfall, with potentially degrading effects on domestic food gardens and local production that supply the urban region, increasing prices and hindering access to healthy food without contaminants or pesticides.

Additionally, medium- and long-term effects include deteriorating the food environment due to the loss of vegetable gardens and crops, exacerbating economic hardship for affected families. Mental health declines as residents face ongoing stress from the trauma of past flooding and the recurring fear of extreme rainfall each year. Reduced physical activity results from the degradation of public spaces or the increased time constraints imposed by daily survival challenges. Furthermore, the risk of infectious diseases rises due to the overburdening of the sewage system and the contamination of drinking water (19, 36).

Interestingly, despite being asked repeatedly, participants did not explicitly mention NCDs. Physical activity was only mentioned when directly asked for, and participants seemed confused by the question as if this was not part of the matter. This indicates that the connections between climate change, climate risks, and events are not clearly connected to NCD risks, aggravation and incidence in people’s mental models.

These findings suggest that both resources and visibility, as highlighted in the homonymous loop, are interconnected elements and play an essential role as drivers in catalyzing responses to climate disasters, as well as articulation between actors as key drivers in the BH climate resilience system. Enhancing the visibility of climate-related challenges, particularly the impacts of heavy rainfall, can increase public awareness and drive collective action. This, in turn, can accelerate resource mobilization, strengthen governmental engagement, and support targeted interventions to mitigate the negative consequences of extreme weather events. A potential unintended consequence is the diminished visibility, resource allocation, and collective action once the strategies successfully address the impacts of heavy rainfall. If left unmitigated, this unintended consequence can result in detrimental cycles of (dis)engagement and (dis)investment, as observed in the system archetype known as ‘drifting goals’ (37).

Participants highlighted the importance of financial support on resources for building resilience actions since new solutions tend to have a higher cost than outdated orthodox solutions, often prevailing in public power decisions. They highlighted the need for transparency in resource allocation and social control to guarantee that public investments are on risk preventive actions, such as urban zoning and planning for climate resilience, restriction of specific constructions, supervision of occupation in risk areas, risk mitigation actions building retaining walls on slopes, parks, urban agriculture, and other nature-based solutions, retention basins, among others. Nevertheless, if misused, both resources and visibility may divert attention and efforts away from addressing deeper structural issues (38).

Successful examples of effective resource allocation, coordinated government action, and collective efforts in Belo Horizonte include the Risk and Disaster Management Group (GGRD - Grupo de Gestão de Riscos e Desastres) (39), as well as urban agriculture initiatives and the public policies that support them. The GGRD holds regular meetings and operates actively during the rainy season (October to March) to prevent climate risks and respond to climate disasters. It brings together public managers from the City Hall and various state agencies, including the Military Police, Fire Department, and utility companies for energy, water, natural gas, and highways, alongside community associations, public and private organizations, and researchers, all under the leadership of Civil Defense. Urban agriculture was indicated as an example of strategic intervention for climate resilience, both as a green solution for slopes that cannot be inhabited to incorporate more permeability in urban areas while also contributing to food security and sovereignty in vulnerable populations. In Belo Horizonte, urban agriculture exists as a spontaneous practice and as structured production units integrated into municipal food security policies. These units operate as a subsidized initiative, receiving technical support from the municipality (40–42).

According to the participants and, as depicted in the Collective action for health loop, volunteers are at the forefront of actions to combat disasters. The emergency volunteer workforce in crisis events is essential, helping to rescue affected families, also offering logistics and material support, cargo and traction vehicles (such as tractors and trucks) to clear roads or load movable assets of affected houses, and offering support at meeting points and referral to shelters and related public services where they may help to cook, clean, organize, provide health and psychological care, and offer comfort for people victim of the disasters. Volunteers also work to properly direct donations and develop strategies to save the material assets of those affected by floods, including preventing vandalism while the person is away from home. Also, donating medicines, food, clothing, and other items to rebuild homes is an essential part of resilience strategies in the face of disasters. All these actions benefit from strategic visibility and resource allocation. However, participants highlight that while volunteers provide crucial immediate support, the scale of many disaster events often far exceeds the available volunteer workforce, and disaster response cannot depend solely on them. They claim it is essential to have a well-structured, institutional response that leads and articulates with volunteer efforts to ensure a more sustainable and coordinated approach (43).

Findings further suggest that community resilience strategies to increase health and decrease vulnerability in the face of climate change in BH could benefit from strong incentives for education and training interventions. The Resilience in action loop underscores this connection by linking the ‘Building resilience’ and the ‘Education’ themes. It highlights the interplay between community preparedness, education, and the essential resources—cultural, social, or material—needed for meaningful participation (44).

Participants’ reflections during workshops emphasized the importance of collective learning and intersectoral collaboration. One crucial aspect mentioned was the need to acknowledge and incorporate popular knowledge masters from *quilombolas* (African descent traditional people), indigenous people, and local communities into decision-making spaces in interaction with public authorities, valuing and incorporating traditional knowledge, practice, and resilience strategies (45).

Climate change education, especially in schools, is vital for empowering younger generations with the knowledge and skills needed to address current and future challenges. Moreover, engaging diverse audiences, including policymakers, is crucial to subsidizing informed decision-making and integrating resilience strategies into public policy, fostering a more climate-aware society at all levels. In this direction, participants highlighted the need for more education, training, and capacity building for public representatives, committees, councils, managers, and institutions, inside and outside public bodies, on climate resilience. According to them, important themes to consider are health, environment, food security, extreme weather events, and equity and empathy when dealing with affected communities. Investing in education for diverse community actors not only equips communities to respond effectively to climate events but also fosters a culture of resilience that permeates various sectors (46).

Another key intervention point raised by participants involves fostering the feeling of belonging to a neighborhood, which creates and sustains engagement in community actions. For example, public spaces considered by residents to be beautiful and pleasant may encourage them to maintain and use these spaces, avoiding degradation and pollution with garbage and rubble and likely engaging in more physical activity (28, 47). Waste thrown on the streets may worsen flooding by clogging rainwater drainage, as highlighted by one participant. As a way of changing the relationship between residents and the environment and collective spaces, they indicated that community strategies such as *Favela Bela* could be leveraged to engage children in activities where they would learn about waste management together with different artistic techniques (48). Another successful initiative mentioned by participants in BH is the *Rede Lixo Zero* (Zero Waste Network), which consists of a consumer network that sustains adequate recycling material collection, helping avoid waste of material and supporting a waste collectors’ association. They also recycle organic waste that is collected in buckets, and the *Rede Lixo Zero* produces fertilizer, avoiding the accumulation of material that would potentially attract venomous animals and disease-vectors (49). These strategies are also seen as health-promoting and climate resilience building, exemplary to the BH context but also to other Global South contexts.

Reflecting on the experience of implementing this study in BH and other cities, we illustrate the potential of including diverse perspectives through participatory approaches like Community-based System Dynamics. For community actors, our results suggest that this may foster effective intervention development, trust, long-lasting intersectoral collaboration, and insights on system intervention points that were not acknowledged by the participants before the discussions (50, 51).

Spontaneous reports from participants evaluating the workshops indicated that moments of exchange with people from other sectors and diverse experiences were highly valued and allowed them “to leave their place and put oneself in someone else’s shoes,” or “think from another perspective,” as it was expected.

By engaging in in-depth discussions on themes, concepts, and situations that are part of daily living, aiming to design a shared mental model, participants may share and be confronted by other perspectives over contentious issues in a safe space where power dynamics are balanced by good moderation, and there is room for dissent and broadening perspectives. This process may generate new insights, foster empathy, trust, and collaboration (9, 51, 52), which is key to a collaborative intersectoral engagement and climate resilience strategies co-creation (9, 51, 52).

We further demonstrate the benefits of global collaborative efforts to engender shared learning between cities and of Community-based System Dynamics as a useful approach in our contexts (22, 50, 51). The collaboration on adapting this approach and implementing the process in different sites was a successful South–South learning, co-creation, and capacity-building experience. It proves that despite cultural differences and long distances, we have much to learn from each other, and research projects that include multiple Global South sites may benefit from this diversity. Despite climate and health being critical global concerns, they become especially pressing in regions where socio-economic inequalities prevail.

Discussing themes, variables, concepts, and connections between our different sites has proven to be a challenge and an experience of mental model actualization in itself since we had to confront pre-conceived ideas based on our local contexts to agree on key approaches that would cross-cut our local implementation of methods. Language and cultural translation of terms such as community, vulnerabilities, and intersectionality, among others, were raised. Specificities regarding the length of the workshop or internet connectivity issues had to be dealt with, and different adaptations were made in each site to cope with the local context and partners’ needs. These apparent challenges only reinforced the fact that rigorous science and genuine knowledge co-creation can take many forms. Yet, even within the diverse contexts of the Global South—both in research and everyday life—it is possible to generate reproducible, meaningful, and widely relatable outcomes and experiences.

Researchers also benefit from the process because their participation is not intended to be merely objective. The first diagram created after the thematic analysis, when presented to the community actors and put into discussion, made it clear that some assumptions and syntheses made by the research team were not accurate and departed from a researcher-biased perspective. When opening the discussion and debating variables and themes’ concepts and connections, the researchers also had the opportunity to broaden their mental models and learn and co-create with the participants’ diverse experiences. By moderating the process and facilitating discussions, researchers also provided their input, so their (our) perspectives are also reflected in the final diagram. Far from being a flaw, this is a strength of the method, as academia is a key actor in collaboratively building local climate resilience strategies. Sharing a common understanding of our cities’ pressing challenges enhances the collective capacity to address these issues effectively.

### 4.1 Limitations

The primary limitation of this study stems from the complex, multifaceted, intersectoral, and dynamic nature of the issue at hand, which makes it difficult to capture its full scope within a single model. However, while models inherently simplify reality, they can still serve as valuable tools for guiding action and decision-making (53). The method acknowledges this limitation, and as a participatory co-creation tool, it depicts an image of the system as understood by the participants at that moment. This recognition allows for reflexivity and methodological coherence, augmenting the value of our results.

Also, despite making great effort to include a diverse group of stakeholders, representatives of the community actors in resilience strategies in BH, and cover different perspectives, we acknowledge that other perspectives may not have been included, which would change the understanding of how the system is designed.

This process was developed within the framework of a funded project, which provided the time and resources necessary to ensure its feasibility. While this may present challenges in terms of scalability and transferability to different local contexts, several aspects of the process suggest its adaptability. Most meetings were conducted online, in-person sessions leveraged existing infrastructure of the local research team, and the modeling software used was available in free versions—or could even be replicated using pen and paper when internet access was limited. These features indicate that CBSD methods can be flexibly adapted to suit the specific conditions and resources of diverse settings.

## 5 Conclusion

We set out to leverage a global network for shared learning between four Global South cities, looking into community climate resilience strategies for each context-specific climate event.

To achieve this, we adopted the Community-based System Dynamics participatory approach, rooted in systems thinking, to identify key strategies, drivers, underlying conditions, and the interconnections shaping their effectiveness. Together with participants, we developed a shared model and highlighted feedback loops that indicate key dynamics in the system to foster climate resilience and health. The final causal loop diagram in Belo Horizonte helps to understand the syndemic of climate change risks and disasters, health effects, and socio-economic inequities, as well as the related climate resilience strategies in place and how to support or enhance them. By establishing this shared mental model, our findings aim to generate actionable insights for policymakers and practitioners, facilitate the identification of key intervention points, and create a foundation for intersectoral engagement and collaboration.

Political and institutional change is not only a matter of data availability or evidences to orient decision-making processes. It is shaped by local governance dynamics and context-specific windows of opportunity, which are influenced by a range of factors beyond the scope of this paper. The potential for transfer and implementation will need to be assessed in each setting through collaborative engagement with participants and the research team.

Ultimately, this work underscores the importance of participatory, system-based approaches in shaping more effective, inclusive, and sustainable climate resilience strategies.

## Data Availability

The original contributions presented in the study are included in the article/Supplementary material, further inquiries can be directed to the corresponding author/s.
