# Supplementary material for: Collective learning for resilience in Global South cities: a community-based systems mapping approach to integrated climate and health action
Source: Front Public Health. 2025 May 19;13:1582550. doi: 10.3389/fpubh.2025.1582550 (PMC12127423; doi:10.3389/fpubh.2025.1582550)
Supplement: Supplementary file 1 [file Data_Sheet_1.docx]

**INTERVIEW GUIDE: COMMUNITY ACTORS**

**Investigating community resilience to climate events risks and disasters**

STRUCTURE OF THE GUIDE

Part 1: Build interviewee profile

Part 2: Elicit the variables

Part 3: Elicit the relationships between variables

Part 4: Review of the diagram

Introductory approach with interviewees:

Hello XXX,

As you know, we are part of an international project which is investigating, among other things, how communities organize strategies to cope with climate change events. We now invite you to contribute to this research by helping us build a board where we will draw links between these strategies and factors and variables that affect or interact with them. It should last between 30 min and one hour, we will ask a couple of questions about you, so we can know you a little better, and the questions about the strategies, while we build this board.

Are you willing to participate? If yes, can I record the meeting, for research purposes, guaranteeing that your name and personal information will not be used?

Thank you for availability and understanding. If you have any questions you can ask me at any point, and if anything makes you uncomfortable you are always free to stop and quit the interview.

Do you have any questions by now?

If not, let’s start with the interview.

| PART 1 Interviewee profile |
| --- |
| - Please explain your role in your organisation, length of time, experience, and profession (if appropriate) - Can you tell me a little bit about your experience working with communities affected by climate risks and disasters in your city? - Have you been directly involved in response to climate events in your community in the past? - Have you ever heard the term complex systems before? If yes, what does it mean to you? - Have you ever heard the term adaptive community response to disasters? If yes, what does it mean to you? |

| Part 2 Elicit the variables |
| --- |
| Place the investigation in the context related climate events, risks and disasters:   - Based on our previous workshop, (x) disaster events were identified as most impactful in the last 10 years, do you recognize any of them?   Identify different strategies and activities:   - Can you specifically list and describe the (x) most impactful strategies or activities that communities have adopted as an adaptive response to disaster event (x)? - To what extent have these strategies been impactful in responding to disaster events (x)?   Identify drivers and conditions:   - Positive → What are the drivers and conditions that (promote) and (sustain) these adaptive responses (see above)? - Negative → What are the drivers and conditions that (weaken) or (make unfeasible) these adaptive responses (see above)?   Identify expected outputs of strategies and activities:   - What were the expected and actual outcomes of the strategies and activities (stated above)?   Identify expected health implications:   - What were/are the potential health implications of these strategies and activities? - And the potential implications on diet and physical activity in particular? |

| Part 3 Elicit the relationships between variables |
| --- |
| *Note: Interviewee needs to give us the key relationships between (variables mapped on white board). We can now capture feedback loops between variables.*   - How do you think these things affect each other or are linked? *[interviewer shows the factors listed and draft connections and add new factors based on the interviewee’s response]*   *Note: Interviewee needs to think about how acting on diet and physical activity can affect the strategies/activities but also influence drivers/conditions that drive adaptive response to disaster (x)*   - How do addressing or not addressing diet and physical activity after the disaster (x) affect the *strategies/activities* and their *drivers/conditions* in the diagram? *[refer to the diagram to expand or change it if necessary]* |

| Part 4 Review of the diagram |
| --- |
| After structure in part 3 has been elicited:   - Is there anything that you would like to add or change in the diagram to better or more clearly represent your views? *[refer to the diagram to expand or change it if necessary]*   Policy probe:   1. Can you describe any specific policies or initiatives that have been successful in supporting these adaptive responses? 2. How have these policies or initiatives been implemented and what has been their impact on the affected communities?   Upstream factors probe:   1. What factors or opportunities (economic/social/health/political) can you describe that take precedence over public health policies and affect diet patterns and physical activity in the advent of a climate disaster?   Recommendation probe:   1. What do you think are the barriers settling priorities and opportunities for meaningful and health driven responses which promote healthy diet and physical activity patterns in the advent of a climate disaster? 2. What do you see as the key challenges and opportunities for supporting adaptive responses to climate disasters in the future? 3. How can we ensure that communities are better prepared and equipped to deal with these risks in the long term? *(refer to the diagram to expand or change it if necessary)* |
